# Synoviocytes and skin fibroblasts show opposite effects on IL-23 production and IL-23 receptor expression during cell interactions with immune cells

**DOI:** 10.1186/s13075-022-02904-9

**Published:** 2022-09-10

**Authors:** Mélissa Noack, Pierre Miossec

**Affiliations:** Immunogenomics and Inflammation Research Unit, Edouard Herriot Hospital, Hospices Civils de Lyon and University of Lyon, Place d’Arsonval, 69003 Lyon, France

**Keywords:** Cell interactions, Stromal cell origin, IL-23 axis, Treatment response

## Abstract

**Background:**

The IL-23/IL-17 axis is involved in inflammatory diseases including arthritis and psoriasis. However, the response to IL-23 or IL-17 inhibitors is different depending on the disease. The aim was to compare the effects of interactions between immune and stromal cells on the IL-23 axis to understand these differences.

**Methods:**

Peripheral blood mononuclear cells were co-cultured with RA synoviocytes or Pso skin fibroblasts, with or without phytohemagglutinin, IL-23, or anti-IL-23 antibody. Production of IL-6, IL-1β, IL-23, IL-17, IL-12, and IFNγ was measured by ELISA. IL-23 and cytokine receptor gene expression (IL-17RA, IL-17RC, IL-12Rβ1, IL-12Rβ2, and IL-23R) was analyzed by RT-qPCR. IL-12Rβ1 and IL-23R subunits were analyzed by flow cytometry.

**Results:**

The production of IL-6, IL-1β, IL-17, IL-12, and IFNγ with synoviocytes or skin fibroblasts was rather similar, and cell interactions with immune cells increased their production, specifically that of IL-17. A major difference was observed for IL-23. Interactions with synoviocytes but not with skin fibroblasts decreased IL-23 secretion while mRNA level was increased, mainly with synoviocytes, reflecting a major consumption difference. IL-23 addition had only one effect, the increase of IL-17 secretion. Cell activation induced similar effects on cytokine receptor gene expression in co-cultures with synoviocytes or skin fibroblasts. The key difference was the cell interaction effects depending on the stromal cell origin. Interactions with synoviocytes increased the expression of both IL-23 receptor subunits at mRNA levels and IL-23R at the surface expression level while interactions with skin fibroblasts decreased their expression at the mRNA level and had no effect at the surface expression level.

**Conclusion:**

Interactions between immune and stromal cells are crucial in cytokine production and their receptor expression. The origin of stromal cells had a major influence on the production of IL-23 and its receptor expression. Such differences may explain part of the heterogeneity in treatment response.

## Introduction

During chronic inflammation, as in inflammatory arthritis or psoriasis (Pso), immune cells migrate to inflammatory sites, resulting in local interactions with resident stromal cells, synoviocytes, and skin fibroblasts. This immune infiltrate includes Th17 cells and other IL-17-producing T cells [1–4]. Their accumulation is found in psoriatic skin lesions [5] and in the synovial tissue in rheumatoid arthritis (RA), ankylosing spondylitis (AS), and psoriatic arthritis (PsA) [6].

IL-23 is the critical driving force behind the Th17 phenotype [7] by amplifying and stabilizing Th17 cell proliferation, promoting pathogenicity and IL-17 secretion. IL-17 stimulates pro-inflammatory cytokine production by several cell types, including synoviocytes and skin fibroblasts [8]. Despite the apparent shared involvement of the IL-23/IL-17 axis, the response to IL-23 or IL-17 inhibitors is different depending on the disease. The most obvious difference is the impressive efficacy of IL-17 and IL-23 inhibition in Pso and to a lower extent in PsA, while only IL-17 inhibition acts in AS and with somewhat negative results in RA [9, 10]. In this study, we selected disease site-associated stromal cells from the two extremes: Pso vs. RA.

Stromal cells located at different anatomic sites contribute to a different clinical distribution, joints for RA and PsA, or skin for Pso. Using an in vitro co-culture model, we have shown that interactions with stromal cells from synovium or skin are critical to amplify pro-inflammatory cytokine production, with a major effect on IL-17 [11, 12].

Despite some similarities, differences are observed, such as the monocyte contribution in interactions with skin fibroblasts but not with synoviocytes [11, 12]. Stromal cells display strong heterogeneity, organ-dependent and location-dependent in a specific organ that is well-described for RA synoviocytes [13]. Synoviocyte subsets have been identified with anatomically discrete, functionally non-overlapping functions on inflammation [14]. This heterogeneity may influence the outcomes of interactions with immune cells and have important implications for the understanding of treatment response.

The aim of this study was to compare the effects of interactions between immune cells and different stromal cells on IL-23. We selected RA synoviocytes and Pso skin fibroblasts, as these cells come from two diseases giving totally opposite responses to the inhibition of the IL-17/IL-23 pathway. Using a co-culture system between synoviocytes or skin fibroblasts and peripheral blood mononuclear cells (PBMCs), we analyzed the effects of cell interactions on cytokine production and receptor gene expression with a focus on IL-23.

## Materials and methods

### Samples

Synoviocytes were obtained from the synovial tissue of RA patients undergoing joint surgery [15]. Skin fibroblasts were obtained from skin biopsies of psoriatic patients who fulfilled the Classification Criteria for Psoriasis [12]. Synovial and skin biopsies were treated as described [11, 12]. Synoviocytes and skin fibroblasts were used between passages 4 to 9. PBMCs from healthy donors were isolated by Ficoll-Hypaque (Eurobio, Courtaboeuf, France) centrifugation. All patients signed an informed consent form. The protocol was approved by the Ethics Committee of the Hospitals of Lyon under the number AC-2016-272.

### Co-culture assay

Stromal cells were seeded overnight, and the next day, PBMCs were added at a 5:1 ratio, with or without phytohemagglutinin (PHA, 5 μg/ml), as described [11, 12]. Treatments, IL-23 (50 ng/ml), anti-IL-23 antibody (1 μg/ml) (R&D Systems, Minneapolis, USA), or control irrelevant antibody (1μg/ml) (Dendritics, Lyon, France) were added to cell cultures, cells alone or co-cultures. After 48 h, supernatants were collected for the analysis and cells for CD3, CD4, CD69, and CD86 staining.

For mRNA expression, co-cultures were initiated by seeding synoviocytes or skin fibroblasts overnight in 12-well plates at a density of 15 × 10^4^ cells/well in a complete RPMI 1640 medium (Eurobio, Courtaboeuf, France, RPMI medium supplemented with 10% fetal bovine serum (FBS), 2 mM l-glutamine, and 100U/ml penicillin/streptomycin). The next day, PBMCs (75 × 10^4^ cells/well) were seeded in a complete RPMI medium, without or with PHA (5 μg/ml) or IL-23 (50 ng/ml). After 24 h, cells were recovered for RNA extraction.

### Flow cytometry

EFluor 450-CD3 (Thermo Fisher Scientific, Waltham, MA, USA, Invitrogen), PE-Cy7-CD4 (Thermo Fisher Scientific, Invitrogen), FITC-CD69 (Becton Dickinson (BD), Franklin Lakes, NJ, USA), PE-CD86 (BD Biosciences), PE-Vio770-CD45 (Miltenyi, Bergisch Gladbach, Germany), PE-IL-12Rβ1 (R&D Systems), and PerCP-IL-23 (R&D Systems) were used for cell surface staining. Cells were incubated for 20 min at 4 °C in staining buffer (PBS 1× + 2% of FBS), washed, and analyzed by flow cytometry (Navios, Beckman Coulter, Brea, CA, USA). Analysis was done with the FlowJo software.

### RNA extraction and real-time qPCR

Total RNA of cells was extracted using the RNeasy Mini Kit (Qiagen®, Hilden, GE) and quantified with the Qubit assay (Invitrogen™, Carlsbad, CA, USA). cDNA was synthesized using the QuantiTect reverse transcription kit (Qiagen®). SYBR green-based real-time qRT-PCRs were performed on the CFX96 Real-Time PCR Detection System (BioRad, Hercules, CA, USA) using the QuantiFast SYBR green kit. Cycle threshold values were normalized with respect to GAPDH. The relative expression of IL-17RA, IL-17RC, IL-23R, IL-12Rβ1, IL-12β2, and IL-23 genes was determined using the comparative threshold cycle method. IL-23R, IL-12Rβ1, and IL-12Rβ2 primers were designed as previously described [13] (Thermo Fischer Scientific, Waltham, MA, USA). GAPDH, IL-17RA, IL-17RC, and IL-23 were QuantiTect Primers (QT01192646, QT00022414, QT00022498, QT00204078, respectively, Qiagen®).

### Enzyme-linked immunosorbent assays (ELISA)

IL-17A, IL-6, IL-1β, IL-12 (Diaclone, Besançon, France), IL-23, and IFNγ (R&D system) productions were evaluated from culture supernatants with commercially available ELISA kits.

### Statistical analysis

Statistical analyses were performed using a paired nonparametric Wilcoxon or Mann-Whitney tests. All analyses were performed with the GraphPad Prism 6 software. *p* values less than 0.05 were considered as significant.

## Results

### Effects of interactions with synoviocytes on Th17/Th1 cytokine production

Physical interactions between stromal cells, such as synoviocytes or skin fibroblasts, and immune cells are critical to induce cytokine production at the site of inflammation in arthritis or in psoriatic skin [11, 12]. The use of a transwell system, preventing direct cell-cell contact but allowing the exchange of soluble factors, confirmed this role, as the cytokine production significantly decreased without physical interactions [11, 12]. Here, we used a co-culture system between stromal cells, RA synoviocytes or Pso skin fibroblasts, and immune cells to evaluate the production of Th1 and Th17 cytokines, with a specific interest in IL-23. RA and Pso stromal cells were selected as these cells come from two diseases giving different responses to the IL-17/IL-23 inhibition.

**Fig. 1:**
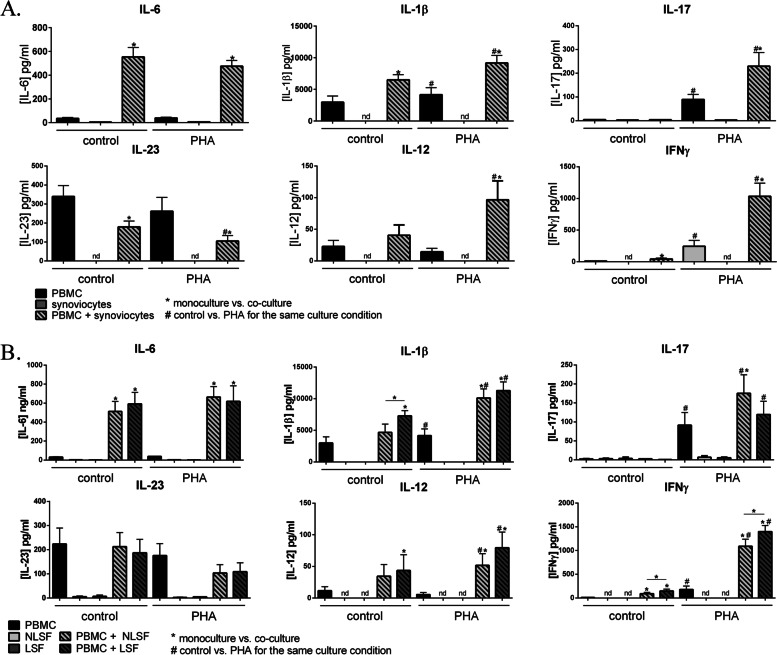
Effects of cell interactions with stromal cells from different origins on Th17/Th1 cytokine production. PBMCs were cultured alone or in co-culture with synoviocytes (**A**) or with non-lesional (NLSF) or lesional skin fibroblasts (LSF) (**B**) at a 5:1 ratio for 48 h, in the presence or absence of PHA (5 μg/ml). Stromal cells (synoviocytes (**A**); skin fibroblasts (**B**)) were also cultured alone for 48 h in the presence or absence of PHA (5μg/ml). Production of IL-6, IL-1β, IL-23, IL-17, IL-12, and IFNγ in cell supernatants was measured by ELISA. “*” compares the condition of culture (PBMCs alone vs. co-culture) in control or in PHA-treated condition. “#” compares the activation state (control or PHA) in the same condition culture. *^#^*p <* 0.05, Wilcoxon test. *n* = 7 experiments for PBMCs and co-cultures and *n* = 6 for stromal cells alone

Cell interactions with RA synoviocytes induced high IL-6 production independently of cell activation compared to cells alone (*p =* 0.016, Fig. 1A). Both cell interactions and activation increased IL-1β secretion, about twofold higher in co-cultures compared to PBMCs alone in control and PHA conditions (*p = 0.*016, Fig. 1A).

We next focused on cytokines of the Th17 pathway, IL-23 and IL-17, and of the Th1 pathway, IL-12 and IFNγ. IL-17 production was almost undetectable in control, PBMCs and co-culture conditions (Fig. 1A). PHA increased IL-17 secretion (*p =* 0.016, Fig. 1A), but its concentration was much higher in co-cultures compared to PBMCs alone (229.7 ± 57.9 vs. 89.7 ± 21.5 pg/ml; *p =* 0.016, Fig. 1A).

Regarding IL-23, cell interactions decreased its production by about twofold compared to PBMCs alone (*p* = 0.016, Fig. 1A). Furthermore, PHA reinforced the IL-23 decrease in co-cultures compared to control (104.8 ± 19.0 vs. 179.5 ± 30.6 pg/ml; *p* = 0.016; Fig. 1A). This decrease in IL-23 secretion could be explained by consumption of IL-23 that induced IL-17, which explained higher IL-17 production in co-culture.

The results for IL-12 were opposed to those for IL-23, with an increase in cell interactions. As for IL-17, the highest production was obtained in activated co-culture condition (*p* = 0.016; Fig. 1A).

IFNγ results were like those of IL-17. In control, IFNγ production was low, even if cell interactions increased this secretion (*p* = 0.016; Fig. 1A). PHA strongly increased IFNγ secretion, but levels were even higher in co-cultures compared to PBMCs alone (1034.3 ± 206.5 vs. 244.6 ± 93.8 pg/ml; *p =* 0.016, Fig. 1A).

To conclude this part on synoviocytes, cell interactions alone were sufficient to induce IL-6 and IL-1β production, while high IL-17, IL-12, and IFNγ secretion required both cell activation and interactions. In contrast to all other cytokines, IL-23 production decreased by interactions with synoviocytes.

### Effects of interactions with skin fibroblasts on Th17/Th1 cytokine production

To compare joint and skin diseases, we looked at the effect of interactions, between PBMCs and skin fibroblasts, on Th17/Th1 cytokine production. Skin fibroblasts came from two biopsies of the same Pso skin area, one from non-lesional (NLSF) skin and one from lesional (LSF) skin.

IL-6, IL-1β, IL-17, IL-12, and IFNγ productions were like those obtained with synoviocytes (Fig. 1B). Interactions with NLSF or LSF induced a high IL-6 production independently of cell activation (*p* = 0.016, Fig. 1B). Both cell interactions and PHA increased IL-1β secretion (*p* = 0.016; Fig. 1B). IL-17 concentration in the control condition was almost undetectable (Fig. 1B). PHA increased IL-17 production in PBMCs and in co-cultures (*p* = 0.016; Fig. 1B), but the highest concentration was observed in activated co-cultures (*p* = 0.016; Fig. 1B). IL-12 secretion was increased by cell interactions, mainly with PHA (*p* = 0.016, Fig. 1B). Cell activation and interactions were sufficient to induce IFNγ production, and its highest concentration was obtained in activated co-cultures (*p* = 0.016; Fig. 1B). It should also be noted that the production of IFNγ was higher with LSF than with NLSF (*p* = 0.016; Fig. 1B).

**Fig. 2:**
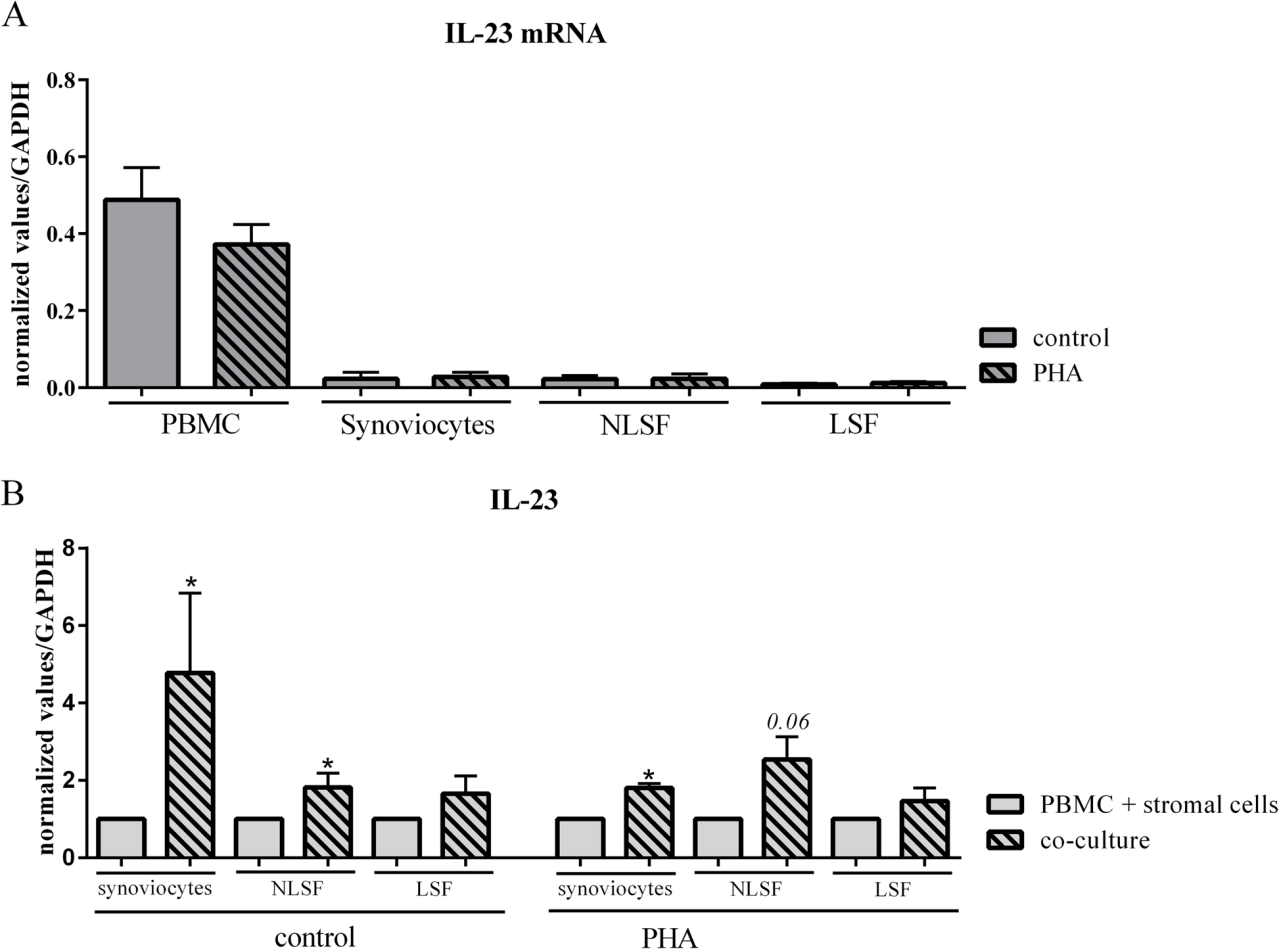
Effects of cell interactions with stromal cells from different origins on IL-23 mRNA expression. PBMCs, synoviocytes, and non-lesional and lesional skin fibroblasts were cultured alone or in co-culture at a 5:1 ratio for 24 h, in the presence or not of PHA (5 μg/ml). At 24 h, RNA of cells alone and co-culture were recovered, and PBMCs + synoviocytes or PBMCs + skin fibroblasts, cultured alone, were pooled to recover RNA. IL-23 expression was analyzed by RT-qPCR. The results showed the gene expression normalized by GAPDH expression. In **B**, the expression in PBMCs + stromal cells was normalized to 1, and the expression in co-cultures was rationalized accordingly. “*” compares the co-cultures vs. PBMCs + stromal cells, *p<0.05, Wilcoxon test. *n* = 5 experiments

The results regarding IL-23 were different than those with synoviocytes, where IL-23 production decreased (Fig. 1A). Such decrease was not observed with skin fibroblasts, either NLSF or LSF, in control or with PHA conditions (Fig. 1B). In order to better understand this difference, we looked at the expression of IL-23 mRNA (Fig. 2). Firstly, as expected, in cells alone, IL-23 mRNA was mainly detected in PBMC (Fig. 2A) and PHA slightly decreased IL-23 mRNA (Fig. 2A) that correlated with the effect on IL-23 production (Fig. 1). In order to observe the effect of cell interactions on IL-23 mRNA, PBMCs and synoviocytes or PBMCs and skin fibroblasts, which had been cultured alone, were pooled, and then RNA was recovered from the combined cell subsets to be compared to the RNA recovered from co-cultures (Fig. 2B). Furthermore, to better compare the different cell types, the expression of cells cultured alone and pooled was normalized to 1 and he expression in the co-culture rationalized accordingly. Cell interactions with synoviocytes significantly increased IL-23 expression in control and PHA (*p* = 0.03, Fig. 2B). With NLSF, in control, the expression was significantly increased (*p* = 0.03, Fig. 2B), and also in PHA, but without reaching a significant value (*p* = 0.06, Fig. 2B). For LSF, it was only a tendency to increase as nothing was significant. The increase of IL-23 mRNA was the highest with synoviocytes (Fig. 2B), while IL-23 production significantly decreased during interactions with synoviocytes but not with skin fibroblasts (Fig. 1). This could involve a higher consumption of IL-23 in co-cultures with synoviocytes compared to skin fibroblasts.

To conclude this part on skin fibroblasts, the results for most cytokines were rather like those with synoviocytes, where interactions induced pro-inflammatory cytokines, mainly resulting from cell activation. On average, LSF were more potent than NLSF. However, a major difference was observed with IL-23. Cell interactions with RA synoviocytes but not with Pso skin fibroblasts decreased IL-23 secretion while IL-23 mRNA was increased with cell interactions. This could reflect a different consumption of IL-23 between co-cultures depending on the stromal cell origin.

### Effects of cell interactions with stromal cells from different origins on cell activation

In order to further investigate the role of stromal cells on PBMC activation, we looked at the expression of activation markers (CD69 and CD86) by flow cytometry. CD3 and CD4 markers were used to distinguish the different PBMC subpopulations.

**Fig. 3:**
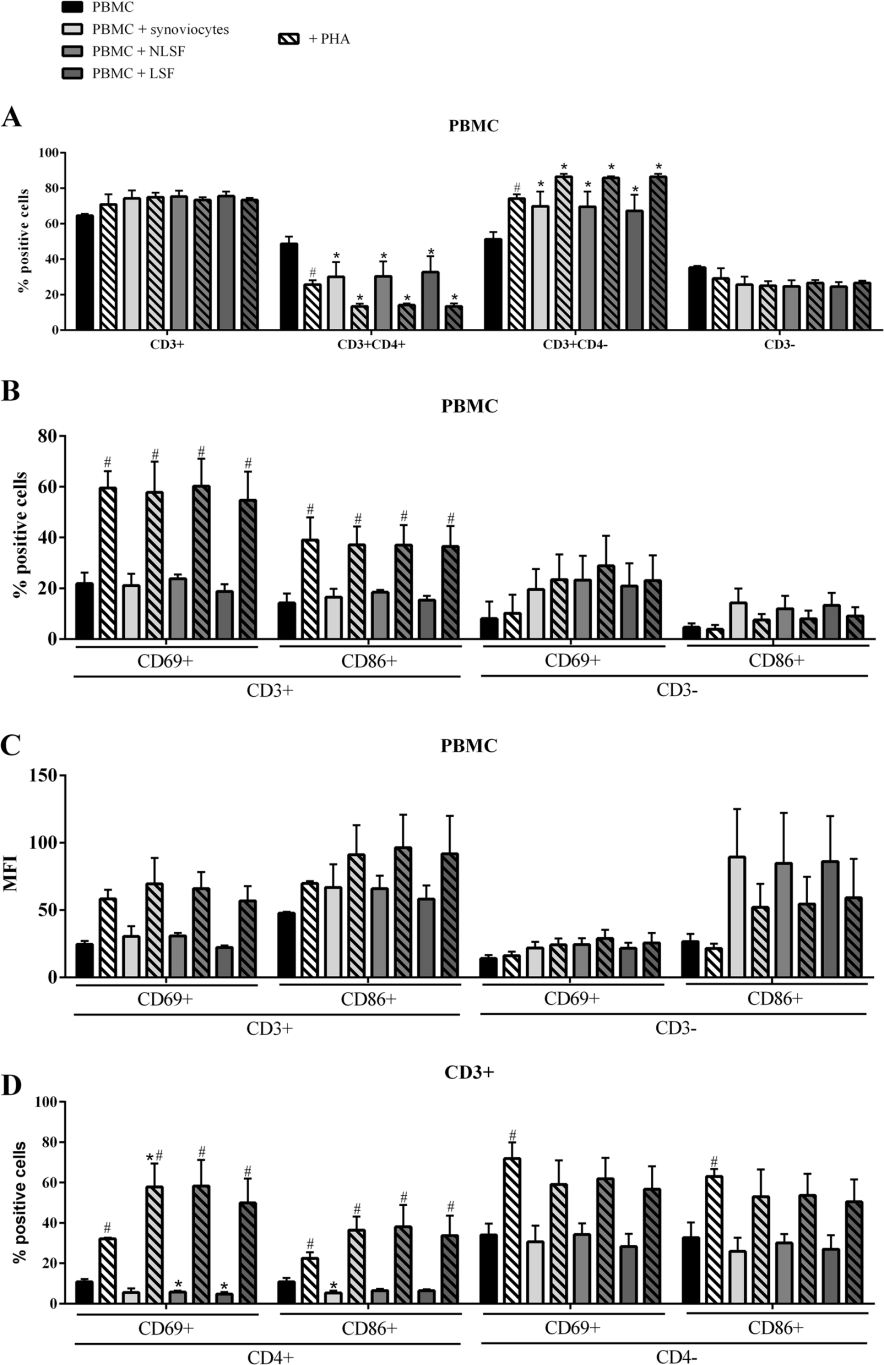
**A**–**D** Effects of cell interactions with stromal cells from different origins on activation markers. PBMCs were cultured alone or in co-culture with synoviocytes or with non-lesional (NLSF) or lesional skin fibroblasts (LSF) at a 5:1 ratio for 48 h, in the presence or absence of PHA (5 μg/ml). At 48 h, cells were recovered and stained for CD3, CD4, CD69, and CD86 before cytometer analysis. The gate was done on PBMCs. “*” compares the condition of culture (PBMCs alone vs. co-culture) in control or in PHA-treated condition. “#” compares the activation state (control or PHA) in the same condition culture. *^#^*p <* 0.05, Mann-Whitney test. *n* = 3 experiments

Firstly, we observed that neither PHA nor cell interactions had an effect on the percentage of CD3+ cells (Fig. 3A). Cell activation by PHA significantly decreased the percentage of CD3+CD4+ cells in PBMCs (*p* = 0.05, Fig. 3A) and trended to diminish this percentage in co-cultures (Fig. 3A). In addition, cell interactions also decreased the percentage of CD3+CD4+ cells, independently of stromal cell origin, compared to PBMCs alone (*p* = 0.05, Fig. 3A).

Then, we looked at CD69 and CD86 activation markers. In CD3+ cells, PHA increased significantly both activation markers (*p* = 0.05, Fig. 3B) with no additional effect of cell interactions (Fig. 3B). In CD3− cells, PHA had no clear effect while cell interactions tended to increase CD69 and in a lower extent CD86 (Fig. 3B). Interestingly, if cell interactions had no clear effect on the percentage of CD69+ and CD86+ cells, they seemed to increase the measurement of fluorescence intensity (MFI), mainly for CD86 (Fig. 3C). In CD3+ cells, we have distinguished CD4+ from CD4− cells (Fig. 3D). In CD4+ cells, PHA increased significantly CD69 and CD86 markers (*p* = 0.05, Fig. 3D), and this was reinforced by cell interactions (Fig. 3D). In CD4− cells, only PHA had an effect, and this reached a significant value in PBMCs (*p* = 0.05, Fig. 3D). The results were the same between synoviocytes and skin fibroblasts.

To conclude, PHA activated mainly CD3+ cells, thus lymphocyte population and cell interactions reinforced this effect mainly in CD4+ lymphocytes. This increased activation of CD3+CD4+ correlated with high IL-17 production requiring PHA activation and cell interactions (Fig. 1).

### IL-23 contribution to Th17/Th1 cytokine production

Based on the differences in cell interactions between synoviocytes and skin fibroblasts on IL-23 production and on clinical results of anti-IL-23 treatment, we next focused on the involvement of IL-23 in cytokine production resulting from these cell interactions, by adding IL-23 or a blocking anti-IL-23 antibody to co-cultures.

**Fig. 4:**
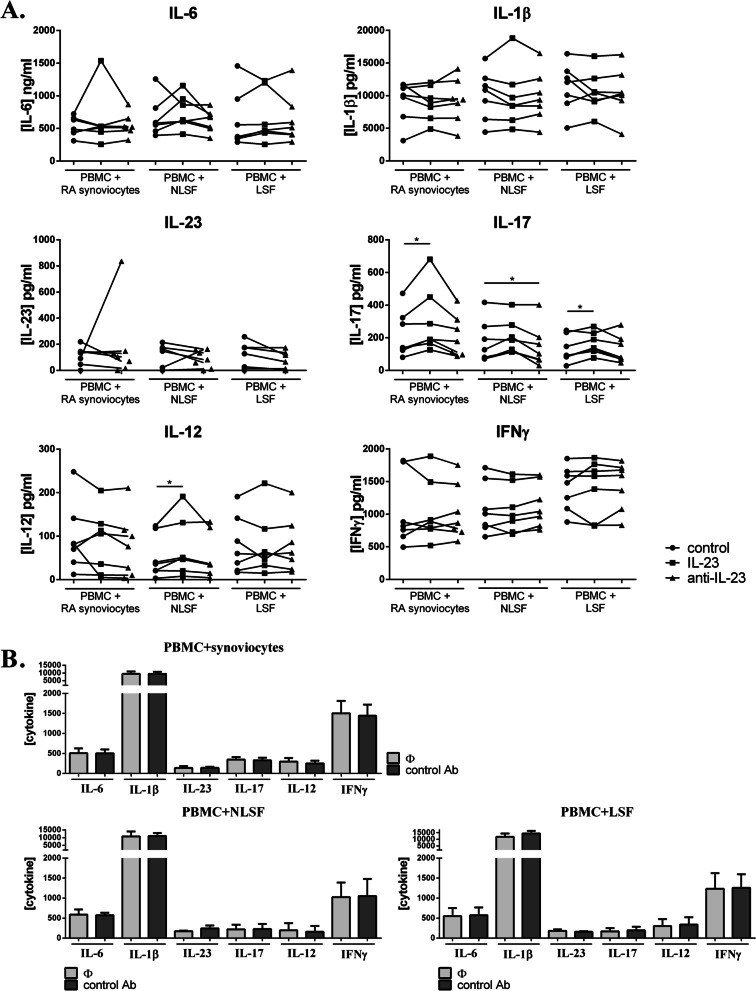
IL-23 contribution to Th17/Th1 cytokine production. PBMCs were cultured in co-culture with synoviocytes or non-lesional (NLSF) or lesional skin fibroblasts (LSF) at a 5:1 ratio for 48 h, in the presence of PHA (5 μg/ml). IL-23 (50 ng/ml) or anti-IL-23 antibody (1 μg/ml) was added in culture (**A**). An antibody control was also used at 1 μg/ml (**B**). Production of IL-6, IL-1β, IL-23, IL-17, IL-12, and IFNγ in cell supernatants was measured by ELISA. “*” compares the condition of culture (IL-23 or anti-IL-23 vs. control). *p < 0.05, Wilcoxon test. *n* = 7 experiments in (**A**) and *n* = 3 in (**B**)

For both synoviocytes and skin fibroblasts, treatment of co-cultures with IL-23 or an anti-IL-23 antibody had no effect on IL-6, IL-1β, or IFNγ (Fig. 4A). With exogenous IL-23 addition, the IL-23 concentration in cultures was around 50 ng/ml (data not shown), reflecting the added IL-23. The anti-IL-23 antibody had no impact on IL-23 production, except for one patient with synoviocytes (control, 92.2 pg/ml vs. anti-IL-23, 836.6 pg/ml), explaining the large SEM (Fig. 4A).

The added IL-23 was functional since IL-17 secretion increased compared to control, in co-culture with synoviocytes and LSF (*p* ≤ 0.03) and to a lower extent with NLSF (Fig. 4A). Conversely, the anti-IL-23 antibody had no effect on co-cultures with synoviocytes or LSF but decreased IL-17 production with NLSF (*p* = 0.02, Fig. 4A). The effect of anti-IL-23 antibody was specific as a control antibody was used in the first experiments, with no effect on cytokine production (Fig. 4B).

Regarding IL-12, IL-23 had an effect only on NLSF co-cultures by increasing IL-12 secretion (*p* = 0.03, Fig. 4A) while the anti-IL-23 antibody had no effect (Fig. 4A).

The effects of exogenous IL-23 or anti-IL-23 antibody were possibly less obvious than expected. Interestingly, the results with synoviocytes and LSF were very similar, but different from those with NLSF, regarding IL-17 and IL-12 production.

### Effect of IL-23 and cell activation on cytokine receptor gene expression in isolated cells

Following the identification of differences between skin fibroblasts and synoviocytes regarding the IL-23 pathway, one key point to consider was a possible difference in cytokine receptor expression. Response to cytokines is mediated by membrane receptors usually made of two chains: IL-17RA/IL-17RC for IL-17, IL-12β1/IL-12β2 for IL-12, and IL-12β1/IL-23R for IL-23. We measured receptor mRNA expression in PBMCs, synoviocytes, or skin fibroblasts alone and in three conditions, control, PHA activation, and IL-23 treatment.

**Fig. 5:**
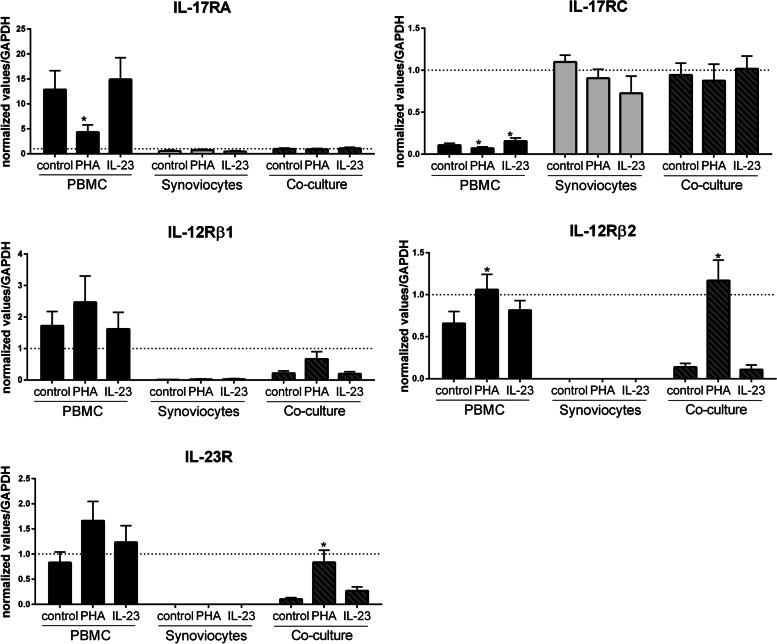
Cytokine receptor gene expression in culture with synoviocytes. PBMCs and synoviocytes were cultured alone or in co-culture at a 5:1 ratio for 24 h, in the presence or absence of PHA (5 μg/ml) or IL-23 (50 ng/ml). RNA was recovered, and RT-qPCR was performed to analyze IL-17RA, IL-17RC, IL-12Rβ1, IL-12Rβ2, and IL-23R expression. The results showed the gene expression normalized to GAPDH expression. “*” compares the condition of culture (PHA or IL-23 vs. control). *p < 0.05, Wilcoxon test. *n* = 6 experiments

**Fig. 6:**
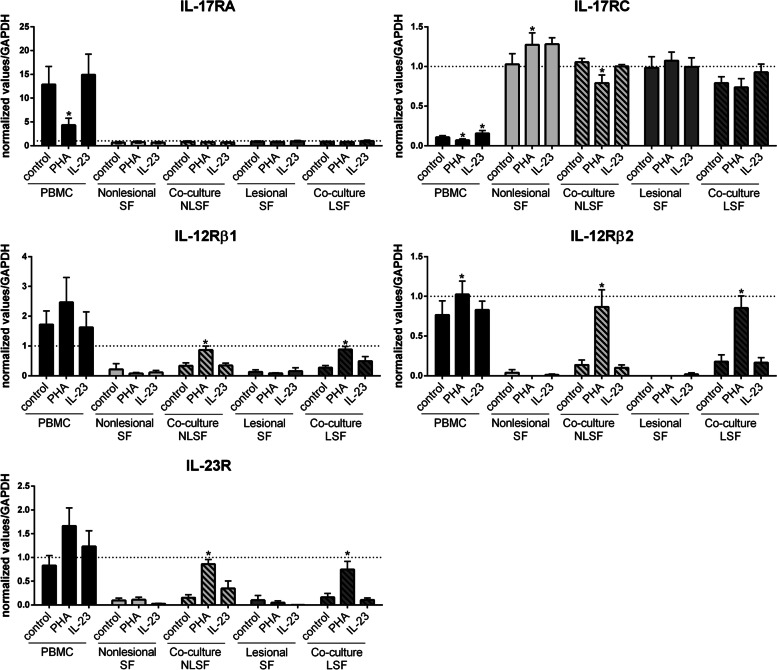
Cytokine receptor gene expression in culture with skin fibroblasts. PBMCs and non-lesional (NLSF) or lesional skin fibroblasts (LSF) were cultured alone or in co-culture at a 5:1 ratio for 24 h, in the presence or absence of PHA (5 μg/ml) or IL-23 (50 ng/ml). RNA was recovered, and RT-qPCR was performed to analyze IL-17RA, IL-17RC, IL-12Rβ1, IL-12Rβ2, and IL-23R expression. The results showed the gene expression normalized by GAPDH expression. “*” compares the condition of culture (PHA or IL-23 vs. control). *p < 0.05, Wilcoxon test. *n* = 6 experiments

In isolated cells, the expression values of all subunits, except IL-17RC, were higher in PBMCs than in synoviocytes (Fig. 5), NLSF, and LSF (Fig. 6). In PBMCs alone, PHA decreased IL-17RA (*p* = 0.03, Figs. 5 and 6) but significantly increased IL-12Rβ2 expression (*p* = 0.03, Figs. 5 and 6). IL-12Rβ1 and IL-23R expression showed a modest increase, which was not significant. IL-23 treatment significantly increased only IL-17RC expression (*p* = 0.03, Figs. 5 and 6).

In stromal cells alone, PHA and IL-23 had no major effect (Figs. 5 and 6), except for a significant increase of IL-17RC expression in NLSF (*p* = 0.03, Fig. 6). In co-cultures, IL-23 had no effect on receptor gene expression while PHA significantly increased IL-12Rβ2 and IL-23R in the three co-cultures with synoviocytes, NLSF and LSF (*p* = 0.03, Figs. 5 and 6). IL-12Rβ1 was significantly increased in co-cultures with NLSF and LSF (*p* = 0.03, Fig. 6), but it was just a tendency with synoviocytes without any significance (Fig. 5).

In conclusion, the expression of these cytokine receptor genes was mainly regulated by PHA activation compared to IL-23, in PBMCs and co-cultures. In stromal cells alone, RA synoviocytes, or Pso skin fibroblasts, receptor gene expression did not seem to be regulated by PHA or IL-23. In PBMCs alone, PHA decreased IL-17RA and IL-17RC expression while it increased IL-12Rβ1, IL-12Rβ2, and IL-23R expression. In co-cultures, PHA similarly regulated receptor gene expression with RA synoviocytes and Pso skin fibroblasts, mainly increasing the expression of IL-23 receptor subunits.

### Effect of cell interactions on cytokine receptor gene expression

**Fig. 7:**
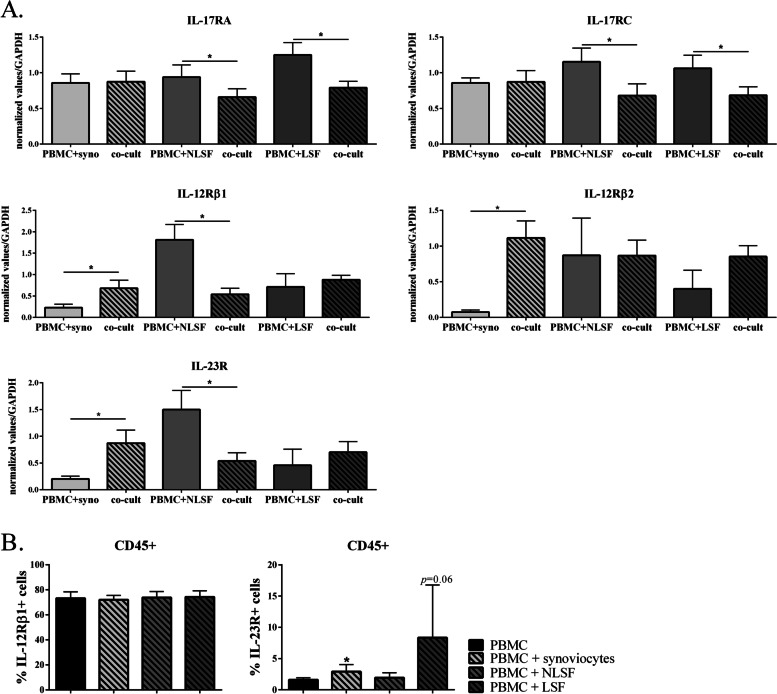
**A**, **B** Effect of cell interactions on cytokine receptor gene expression. In **A**, PBMCs, synoviocytes, and non-lesional (NLSF) and lesional skin fibroblasts (LSF) were cultured alone or in co-culture at a 5:1 ratio for 24 h, in the presence of PHA (5 μg/ml). At 24 h, RNA from co-culture were recovered, and PBMCs + synoviocytes or PBMCs + skin fibroblasts, cultured alone, were pooled to recover RNA. IL-17RA, IL-17RC, IL-12Rβ1, IL-12Rβ2, and IL-23R expression was analyzed by RT-qPCR. The results showed the gene expression normalized by GAPDH expression. “*” compares the condition of culture (co-cultures vs. pooled cells). *p < 0.05, Wilcoxon test. *n* = 6 experiments. In **B**, PBMCs, synoviocytes, and non-lesional (NLSF) and lesional skin fibroblasts (LSF) were cultured alone or in co-culture at a 5:1 ratio for 24 h, in the presence of PHA (5 μg/ml). At 24h, cells were recovered and stained for CD45, IL-12Rβ1, and IL-23R before cytometer analysis. *p < 0.05, Wilcoxon test. *n* = 5 experiments

As cytokine receptor gene expression and regulation were similar in synoviocytes and skin fibroblasts alone, we next focused our interest on the effect of cell interactions. To investigate this point more rigorously, PBMCs and stromal cells were cultured alone or in co-cultures, with PHA. At the end of the culture, PBMCs and synoviocytes or PBMCs and skin fibroblasts, which had been cultured alone, were pooled, and then RNA was recovered from the combined cell subsets. This allowed recovering RNA from both cell types, which we could compare to RNA recovered from co-cultures. Using this protocol, we could observe the direct effect of cell interactions on receptor gene expression (Fig. 7).

Interactions with synoviocytes did not influence IL-17 receptor subunit expression, while, in contrast, the latter decreased with skin fibroblasts, both NLSF and LSF (*p* = 0.03, Fig. 7). This discrepancy between stromal cells was even more pronounced for IL-12Rβ1, IL-12Rβ2, and IL-23R expression. Interactions with synoviocytes increased IL-12Rβ1 and IL-23R expression (*p* ≤ 0.03, Fig. 7), and those with skin fibroblasts strongly decreased it for NLSF (*p* = 0.03, Fig. 7) and had no effect on LSF. IL-12Rβ2 expression was strongly increased in synoviocytes (*p* = 0.03, Fig. 7) while no significant effect was observed with NLSF and LSF. Nevertheless, a slight increase was observed with LSF.

To confirm the difference in IL-23 receptor regulation, we analyzed its expression by flow cytometry. The subunits IL-12Rβ1 and IL-23R were observed in cells alone or in co-cultures. PBMCs and stromal cells were distinguished by CD45, expressed by PBMCs (93.6 ± 6.22% of CD45+ cells in PBMCs alone and 89.1 ± 2.6% in co-cultures, data not shown) but not by stromal cells (less than 1% of positive cells, data not shown). As expected regarding mRNA results, stromal cells did not express the subunits of the IL-23 receptor (< 1% of positive cells, data not shown). In PBMCs, IL-12Rβ1 was constitutively expressed, and its expression was not affected by cell interactions (Fig. 7B). However, the IL-23R subunit was weakly expressed in PBMCs (1.6 ± 0.3% of IL-23R+ cells, Fig. 7B). Its expression was significantly increased by cell interactions with synoviocytes (2.91 ± 1.1% of IL-23R+ cells, *p* = 0.03, Fig. 7B) but not with NLSF (1.9 ± 0.8% of IL-23R+ cells, Fig. 7B). These results reinforced the differences observed at the mRNA level. Moreover, the expression of the receptors was done within 24 h of culture, and it would be interesting to look at later times to see if this difference persists and increases. With LSF, the results were more difficult to interpret because of a large standard deviation due to various responses, including one patient who increased the percentage of IL-23R+ cells to 22.6%. However, what we could note was that the result was finally between synoviocytes and NLSF, as for mRNA, with a tendency to increase IL-23R expression without reaching significance.

To conclude this part, cell interactions regulated receptor gene expression very differently, and even more in an opposite direction, depending on the stromal cell origin. Briefly, interactions with synoviocytes increased the expression of both IL-23 receptor subunits at mRNA level and IL-23R at the surface expression level while interactions with non-lesional skin fibroblasts decreased their expression at the mRNA level and had no effect at the surface expression level. The situation in LSF was in between that of NSLF and synoviocytes.

## Discussion

The IL-23/IL-17 axis is involved in several inflammatory diseases, including RA, PsA, AS, and Pso, among many others. Although their understanding has probably been oversimplified, the clinical reality showed areas where such understanding was too limited to explain the heterogeneity of response to treatment targeting IL-23 vs. IL-17 [9, 10].

Using our tools and previous results, we focused on possible differences in the effect of interactions between immune cells and stromal cells from different origins on the IL-17/IL-23 pathway, with a special interest in IL-23. We have selected disease site-associated stromal cells from the two extremes: Pso vs. RA, with Pso showing sensitivity to IL-17/IL-23 inhibition and RA with poor or no sensitivity.

Cell interactions were sufficient to induce IL-6 and IL-1β secretion. They were also sufficient but further enhanced by cell activation for IFNγ and IL-12 production. High IL-17 secretion required both cell activation and interactions. These results were rather similar with stromal cells from both joints and skin. The major difference was for IL-23 production. Interactions with synoviocytes decreased IL-23 production. This was coherent with the low levels of bioactive IL-23 found in RA synovium [16]. In contrast, IL-23 production was not decreased with skin fibroblasts, in line with the presence of bioactive IL-23 in psoriatic plaques [17]. In addition, we have shown that mRNA was largely increased in co-cultures with RA synoviocytes, while the production significantly decreased. Added to the increased receptor expression, this result correlated with a high early IL-23 consumption, reducing the bioavailability of IL-23. In Pso context, IL-23 mRNA increased to a lower extent in co-cultures compared to PBMCs while the production was almost similar. Added to the decreased receptor expression, this result rather correlated with a weak but constant IL-23 consumption, keeping a certain bioavailability. This reflected an important difference between the two systems. It makes sense that target bioavailability could be associated with a better response [18]. Our results support this hypothesis with synoviocytes reducing IL-23 production leading to a lower response in arthritis compared to skin fibroblasts in Pso. A recent study in PsA that compared paired skin and synovium also supports this concept, with a higher expression of IL-23 in lesional skin compared to PsA synovium [19]. Finally, additional studies are needed to better compare RA synoviocytes versus PsA synoviocytes versus PsA skin fibroblasts or Pso skin fibroblasts. This would provide a more general view of the importance of stromal cells’ origin in the production and thus the bioavailability of IL-23.

Our previous results already identified another difference, regarding the contribution of monocytes to IL-17 secretion. With synoviocytes, monocyte elimination from PBMCs had no impact on IL-17 production [11], in line with the persistent high IL-17 production observed here despite the IL-23 decrease. In contrast, with skin fibroblasts, removal of monocytes and thus of the main source of IL-23 decreased IL-17 production. In this case, monocyte removal acts on a cascade, decreasing first IL-23, then IL-17 secretion [12]. This reflects a key difference in the mechanisms of IL-17 production depending on stromal cell origin. In some conditions, IL-17 production can be IL-23-independent, as suggested for RA, and in part for PsA [20]. These results suggest that interactions between lymphocytes and synoviocytes have a direct role in IL-17 secretion without the need for monocytes and thus for IL-23. In contrast, with skin fibroblasts, monocytes do contribute, at least in part through their IL-23 production. These divergent results are consistent with the hypothesis of potential differences in the IL-23/IL-17 axis between Pso, PsA, and AS and could in part explain the different responses to treatment.

Next, we focused on IL-23 contribution. To some extent, surprisingly, the effects of exogenous IL-23 or anti-IL-23 antibody were less obvious than expected. Interestingly, the results with synoviocytes and LSF were very similar, but different from those with NLSF, regarding IL-17 and IL-12 production. This point highlights that stromal cell heterogeneity is both tissue- and disease-specific. Pathologic changes well studied in RA synoviocytes have been shown to contribute to chronicity through an aggressive and immunostimulatory phenotype [13]. These results remain to be expanded to other anatomic sites and diseases.

The lack of a clear effect of IL-23 inhibition may be due to a kinetic effect, with IL-23 acting early during Th17 differentiation. In our experiments, we blocked IL-23 directly in co-culture, while IL-23 probably acts before cell interactions, possibly even before migration. Kinetic experiments could provide a better understanding of the site of IL-23 action. The kinetics also could contribute to the understanding of the difference in anti-IL-17 effects between Pso and RA. In RA, IL-17 has been demonstrated to act at early stages [21, 22]. Trials addressing this point in early RA are still lacking. In contrast, in Pso, the permanent presence of IL-23 and Th17 cells in lesion plaques leads to a continuous IL-17 secretion and contribution.

To explain some of the differences described above, we then focused on cytokine receptor gene expression in cells involved in the IL-17/IL-23 axis. The interesting results came from the effects of cell interactions. We have shown that cytokine production resulting from cell interactions was similar between synoviocytes and skin fibroblasts for almost all cytokines, except IL-23. The receptor gene expression regulation was found to be more dependent on stromal cell origin. IL-17RA and IL-17RC expression was not affected by interactions with RA synoviocytes but decreased with Pso skin fibroblasts. IL-12Rβ1, IL-12Rβ2, and IL-23R regulation was more complex. The three subunits were regulated in an opposite way in synoviocytes vs. NLSF. Their expression was increased in synoviocytes but decreased in NLSF. The heterogeneity linked to stromal cell origin can thus influence the expression of cytokine receptors and cellular responses. Moreover, the heterogeneity within subsets of stromal cells can generate different responses, as shown by the differences between NLSF and LSF. IL-12Rβ1 expression was not affected by cell interactions with LSF while it was largely decreased with NLSF. In addition, IL-12Rβ2 expression was not affected or slightly decreased with NLSF, but rather slightly increased with LSF. IL-23R expression was also decreased with NLSF, but not with LSF. The difference between NLSF and LSF could come from the pathological state of cells. LSF came from lesional skin thus with an inflammatory state more important than NLSF which came from “healthy” skin. The results with LSF seemed to be between those with NLSF and those with RA synoviocytes. RA synoviocytes display an inflammatory state that could be closer to LSF than NLSF. The inflammatory state of stromal cells, independently of their origin, may influence the regulation of cytokine gene receptor expression. Furthermore, the expression at the cell surface of IL-23R also displayed a different regulation depending on stromal cell origin. While the IL-12Rβ1 subunit was constitutively expressed in PBMCs, mainly in lymphocytes, and not altered by cell interactions, IL-23R subunit was significantly increased by cell interactions with synoviocytes but not with NLSF. As for mRNA, the results for LSF were between synoviocytes and NLSF. As the cell surface expression of the receptors was done at the same time point as mRNA, it would be interesting to look at later times to see if this difference persists and increases, meaning an increase of IL-23R with synoviocytes and no effect or a decrease with NLSF. These results confirm the importance of stromal cell heterogeneity that appears at different levels, tissues, diseases, and pathological states. This heterogeneity has been highlighted in PsA, by comparing matched skin and synovial tissues [19]. This highlights the importance of considering the heterogeneity of stromal cells in the understanding of treatment response. To confirm and extend the results on cytokine receptor expression, more experiments should be done. A transwell system could be used to confirm the critical role of cell interactions in gene expression regulation. The protein level should be also determined to extend the study of gene expression to a more functional level with the receptor expression at the cell surface. These first results allow us to partially explain the differences that appear in the response to IL-17/IL-23 inhibition depending on the disease. However, this study needs to be continued to better understand the mechanisms involved in the differences observed between RA and Pso on the IL-17/IL-23 axis.

## Conclusion

In conclusion, interactions between immune and stromal cells are key contributors to cytokine production but also in the regulation of their receptor expression. These results highlight that both origin and activation state of stromal cells influence the response of cell interactions and even lead to opposite effects on IL-23 production resulting in different effects on the IL-17 axis. The understanding of such differences could provide, at least in part, an explanation for some of the unexpected differences in response to IL-23 vs. IL-17 inhibitors depending on diseases and disease-specific stromal cell subsets.

## Data Availability

The dataset supporting the conclusions of this manuscript is included within the manuscript.

## Abbreviations

AS: Ankylosing spondylitis
ELISA: Enzyme-linked immunosorbent assay
GAPDH: Glyceraldehyde-3-phosphate dehydrogenase
IFNγ: Interferon gamma
IL-: Interleukin-
LSF: Lesional skin fibroblasts
NLSF: Non-lesional skin fibroblasts
PBMC: Peripheral blood mononuclear cells
PHA: Phytohemagglutinin
PsA: Psoriatic arthritis
Pso: Psoriasis
RA: Rheumatoid arthritis
RNA: Ribonucleic acid
RPMI: Roswell Park Memorial Institute
RT-qPCR: Real-time quantitative reverse transcription polymerase chain reaction
Th: Helper T cell
